# Bridging the diagnostic gap: the role of culture methods in the era of syndromic panels for central nervous system infections

**DOI:** 10.3389/fcimb.2026.1853020

**Published:** 2026-05-21

**Authors:** Giuseppe Sberna, Silvia D’Arezzo, Maria Beatrice Valli, Carla Nisii, Eleonora Lalle, Marina Selleri, Fabiano Brillo, Carlo Giacomo Palazzo, Carolina Venditti, Claudia Rotondo, Valentina Antonelli, Fabrizio Maggi, Carla Fontana, INMI Laboratories Group

**Affiliations:** 1 Laboratory of Virology and Laboratories of Biosafety, National Institute for Infectious Diseases Lazzaro Spallanzani - Istituto di Ricovero e Cura a Carattere Scientifico, Rome, Italy; 2 Laboratory of Microbiology and Biological Bank, National Institute for Infectious Diseases Lazzaro Spallanzani - Istituto di Ricovero e Cura a Carattere Scientifico, Rome, Italy

**Keywords:** central nervous system infections, cerebrospinal fluid, encephalitis, meningitis, microbial culture, polymerase chain reaction

## Abstract

**Introduction:**

Syndromic molecular panels have improved the rapid etiological diagnosis of central nervous system (CNS) infections by enabling the simultaneous detection of multiple pathogens directly from cerebrospinal fluid (CSF). Their speed and standardization support early clinical decision−making, particularly in emergency and referral settings. However, the closed architecture of these panels limits pathogen coverage and does not allow antimicrobial susceptibility testing, potentially resulting in incomplete diagnostic assessment. A retrospective real−world evaluation of the BioFire^®^ FilmArray^®^ Meningitis/Encephalitis Panel (FA) was conducted over three years at a national reference center, comparing its performance with conventional culture.

**Methods:**

A total of 2,586 CSF samples with paired molecular and culture results were included. Diagnostic concordance, sensitivity, specificity, and predictive values were calculated using standard statistical methods. Analyses were conducted both on the complete dataset and after restriction to bacterial targets included in the syndromic panel.

**Results:**

Overall, there was moderate agreement between syndromic testing and culture (Cohen’s kappa = 0.61), with the FilmArray panel showing a specificity of 97.5% and a sensitivity of 64.1%. Restricting the analysis to the bacterial targets included in the panel substantially improved the agreement (κ = 0.74), increasing the sensitivity to 93.5% and giving the test a negative predictive value of 99.7%. Importantly, conventional culture identified clinically relevant pathogens not included in the syndromic panel, including *Staphylococcus aureus*, *Streptococcus pyogenes*, and rare environmental organisms, highlighting its essential complementary role.

**Discussion:**

Although syndromic molecular panels represent a valuable diagnostic tool for CNS infections, they cannot be used as standalone assays. Optimal diagnostic accuracy requires systematic integration of syndromic testing with conventional culture to ensure comprehensive pathogen detection and appropriate antimicrobial management. These findings support the need for next−generation multiplex platforms with expanded target coverage informed by real−world epidemiology.

## 1 Introduction

Central nervous system (CNS) infections, including meningitis and encephalitis, require accurate etiological diagnosis because delayed or inappropriate therapy is associated with morbidity and mortality (Kanjilal et al., 2019; van de Beek et al., 2021). Early identification of the causative agent is challenging, as clinical presentations are often non-specific and reflect a spectrum of bacterial, viral, fungal, and mycobacterial pathogens (Kanjilal et al., 2019).

Syndromic molecular panels (SPs) accelerate diagnostic workflows, particularly in emergency and referral settings (Dien Bard and McElvania, 2020; Cassidy et al., 2021). By enabling rapid detection of common meningoencephalitis agents, SPs can support earlier, more informed initial clinical decision-making (Dien Bard and McElvania, 2020; Cassidy et al., 2021).

However, the diagnostic completeness of SPs is intrinsically constrained by their closed target architecture: they only detect organisms included in the panel, and therefore cannot identify clinically relevant pathogens outside the target list (Ramanan et al., 2018; Dien Bard and McElvania, 2020). For example, in the BioFire FilmArray Meningitis/Encephalitis panel (FA) the bacterial targets are limited to *Escherichia coli* K1, *Haemophilus influenzae*, *Listeria monocytogenes*, *Neisseria meningitidis*, *Streptococcus agalactiae* and *Streptococcus pneumoniae*; consequently, important etiologies such as *Staphylococcus aureus* (and therefore MRSA), *Streptococcus pyogenes*, and several yeasts and environmental organisms are not covered by this assay. This limitation is particularly relevant in CNS infections, where rare or emerging pathogens may be responsible for disease (Kanjilal et al., 2019; Riddell and Wheat, 2019; van de Beek et al., 2021). Moreover, pathogens such as *S. aureus* and group A streptococci are well-recognized causes of adult meningitis in specific clinical contexts, including community-acquired and invasive presentations, supporting the need for diagnostic strategies that remain robust beyond predefined panels (Marquardt et al., 2024; Nielsen et al., 2026).

For these reasons, culture-based diagnostics remain essential within an integrated workflow, as they provide broad, unbiased pathogen recovery and enable phenotypic antimicrobial susceptibility testing (AST), capabilities that SPs do not deliver (Ramanan et al., 2018; Kanjilal et al., 2019). In referral centers, where SPs may be requested as stand−alone tests by peripheral hospitals, systematic culture remains essential to mitigate the risk of missed etiologies and to support appropriate targeted therapy, also in the context of incomplete clinical information, including data on ongoing antimicrobial treatment.

This study reports a three−year real−world experience from a national reference center implementing systematic parallel testing of CSFs using FA alongside conventional culture. By retrospectively analyzing paired results collected between 2023 and 2025, this study aimed to (i) quantify the incremental diagnostic contribution of culture, (ii) characterize clinically relevant pathogens missed because they were off−panel of the FA, and (iii) assess the potential clinical implications of relying exclusively on syndromic testing for the diagnosis of CNS infections.

## 2 Materials and methods

The National Institute for Infectious Diseases (INMI) “Lazzaro Spallanzani” IRCCS is a national and international reference center for the diagnosis, treatment, and research of infectious diseases. Since 2018, the INMI Microbiology Laboratory has been designated as the Lazio Regional Reference Centre for the execution of syndromic testing for meningitis and encephalitis within a regional diagnostic protocol. In this role, INMI routinely receives CSFs from hospitals across the region. However, starting in 2023, a combined diagnostic approach integrating molecular syndromic testing and conventional culture was implemented in order to identify clinically relevant pathogens not detected by the SP, including off-panel bacterial and fungal etiologies (e.g., *S. aureus*, *S. pyogenes*, *Histoplasma capsulatum*, *Candida* spp.) (Ena et al., 2021; López et al., 2024).

### 2.1 Specimen collection

Between January 2023 and December 2025, a total of 3,035 CSFs were initially analyzed. Duplicate submissions were excluded, retaining only the first sample per patient per year, as no patient tested positive for different pathogens in multiple samples within the same year. In addition, samples for which culture results were unavailable, primarily due to insufficient CSF volume received by the laboratory, were excluded. The final dataset therefore, comprised 2,586 CSF samples.

For microbiological analysis, a minimum volume of 1.5–2 mL of CSF was collected in a sterile, additive−free tube, in accordance with World Health Organization (WHO) recommendations for timely and accurate diagnosis (WHO). Samples were promptly transported to the laboratory and processed within two hours of collection.

### 2.2 Macroscopic examination

The gross appearance of each CSF was recorded, noting whether it was clear, turbid, blood-stained, or xanthochromic. The presence of fibrin or blood clots was also documented. WHO guidelines recommend noting these features as part of the initial diagnostic assessment standards (WHO).

### 2.3 Culture and microscopy

CSFs were centrifuged at 3000 rpm for 10 minutes, according to WHO guidelines on meningitidis (WHO). After aseptic decanting of the supernatant, the sediment was vortexed, and a drop was used to inoculate chocolate and blood agar plates (Thermo Fisher Scientific, San Diego, CA, USA). Inoculated plates were incubated at 37 °C in 5% CO_2_ until 5 days.

### 2.4 Plate antibiotic residual test

To detect residual antimicrobial activity potentially inhibiting culture growth, a Plate Antibiotic Residual (PAR) test was performed on all FA−positive/culture−negative CSF samples. A 10 µL aliquot of CSF was applied onto PAR-Test agar plate a medium pre-inoculated with spore of *Geobacillus stearothermophilus* ATCC 7953, a reference strain highly susceptible to commonly used antimicrobial agents. Plates were incubated at 55 °C for 18–24 hours. The appearance of a clear inhibition zone around the CSF spot was interpreted as evidence of residual antimicrobial activity capable of suppressing bacterial growth in conventional culture.

### 2.5 Culture examination

Culture plates were inspected for microbial growth. In the absence of visible growth, incubation was prolonged for up to 5 days. When growth was observed, it was semi−quantitatively assessed (scanty), and isolates were subsequently identified and tested for AST. According to WHO recommendations, culture remains the reference standard for the diagnosis of bacterial meningitis, with extended incubation required to detect slow−growing organisms (WHO).

### 2.6 Identification and antimicrobial susceptibility testing of isolates

Isolates obtained by culture were identified using the MALDI−TOF Biotyper Sirius system (Bruker Daltonics, Bremen, Germany) with MBT Compass software (version 4.2). AST was performed using Phoenix panels on the Phoenix automated system (Becton Dickinson Diagnostics, San Jose, CA, USA). Minimum inhibitory concentrations (MICs) were interpreted according to the European Committee on Antimicrobial Susceptibility Testing (EUCAST) guidelines (Clinical breakpoint tables). For isolates for which a dedicated commercial panel was not available, AST was performed manually using the agar diffusion method (Kirby–Bauer or gradient-test), in accordance with EUCAST standards (Clinical breakpoint tables).

### 2.7 Cryptococcal antigen and other tests

Cryptococcal antigen testing was performed, when requested, using a latex agglutination assay (Cryptococcal Antigen Latex Agglutination System; D.I.D. S.p.A., Milan, Italy).

#### 2.7.1 BioFire^®^ FilmArray^®^ Meningitis/Encephalitis (ME) Panel

CSFs from patients with suspected meningitis or encephalitis were analyzed using the BioFire^®^ FilmArray^®^ Meningitis/Encephalitis (ME) Panel (bioMérieux). This multiplex PCR−based assay enables the simultaneous detection of 14 bacterial, viral, and fungal pathogens directly from CSFs.

Briefly, 200 µL of CSF were mixed with the provided sample buffer and loaded into the test pouch together with the hydration solution. The pouch was then inserted into the BioFire^®^ FilmArray^®^ system, where nucleic acid extraction, amplification, detection, and result interpretation were carried out automatically. The entire process required approximately one hour. The test was routinely performed in parallel with conventional microbiological methods and results were subsequently compared with culture findings to assess the diagnostic performance of the assay.

### 2.8 Statistical analysis

Statistical analyses were performed to evaluate the diagnostic performance of the FA in comparison with conventional culture. Sensitivity, specificity, positive predictive value (PPV), and negative predictive value (NPV) were calculated using standard definitions based on the distribution of true positive, false positive, true negative, and false negative results. PPV and NPV were calculated exclusively for bacterial pathogens included in the FA panel. Since viral targets are not the primary focus of this study, they were considered only in the descriptive analysis. Ninety−five percent confidence intervals (95% CI) for binomial proportions were estimated using the exact Clopper–Pearson method, which provides non−asymptotic, distribution−free bounds appropriate for discrete diagnostic outcomes and avoids the limitations associated with normal−approximation intervals (Clopper and Pearson, 1934). Because specificity and NPV approached values close to 1, the McGrath & Burke method was additionally applied to refine uncertainty estimates for “rare−event” binomial proportions by defining the margin of error relative to the magnitude of high−probability estimates (McGrath and Burke, 2024).

To assess inter−method agreement between the FA and culture, Cohen’s kappa (κ) statistic was calculated, together with its 95% CI, as a measure of concordance beyond chance (GraphPad Software, [[NoYear]]). All statistical analyses were performed using validated routines implemented in standard biomedical statistical software.

## 3 Results

To provide an overview of routine diagnostic outputs in a real−world referral setting, an initial unadjusted comparison between FA and culture was performed, including bacterial pathogens not targeted by the FA panel (**Table 1**). To do this, 2,586 CSF samples with paired FA and conventional culture results were included in the analysis. Overall, 2,370 samples were concordantly negative by both methods, indicating a high level of agreement for pathogen exclusion in routine clinical practice (**Table 1**). One hundred specimens were concordantly positive, 56 were FA−negative with culture−positive results, and 60 were FA−positive with culture−negative results (**Table 1**); for the latter group, the PAR test result was positive in 75% of cases. The overall inter-method agreement was moderate, with a Cohen’s κ coefficient of 0.609 (95% CI, 0.544–0.674). Based on the unadjusted contingency table (**Table 1**), the FA showed a sensitivity of 64.1% (95% CI, 56.0–71.6) and a specificity of 97.5% (95% CI, 96.8–98.1%).

**Table 1:** Unadjusted contingency table comparing BioFire® FilmArray® Meningitis/Encephalitis Panel (FA) and conventional culture results in CSFs.

		Biofire® Filmarray® Meningitis/Encephalitis (ME) Panel		
		Positive	Negative	Total
Culture	Positive	100	56	156
	Negative	60	2370	2430
	Total	160	2426	2586

Values reflect real−world laboratory outputs obtained before exclusion of viral infections and bacterial pathogens not included in the FA panel. This table is presented for descriptive purposes only and does not represent intrinsic diagnostic performance of the FA for bacterial targets.

**Figure 1:**
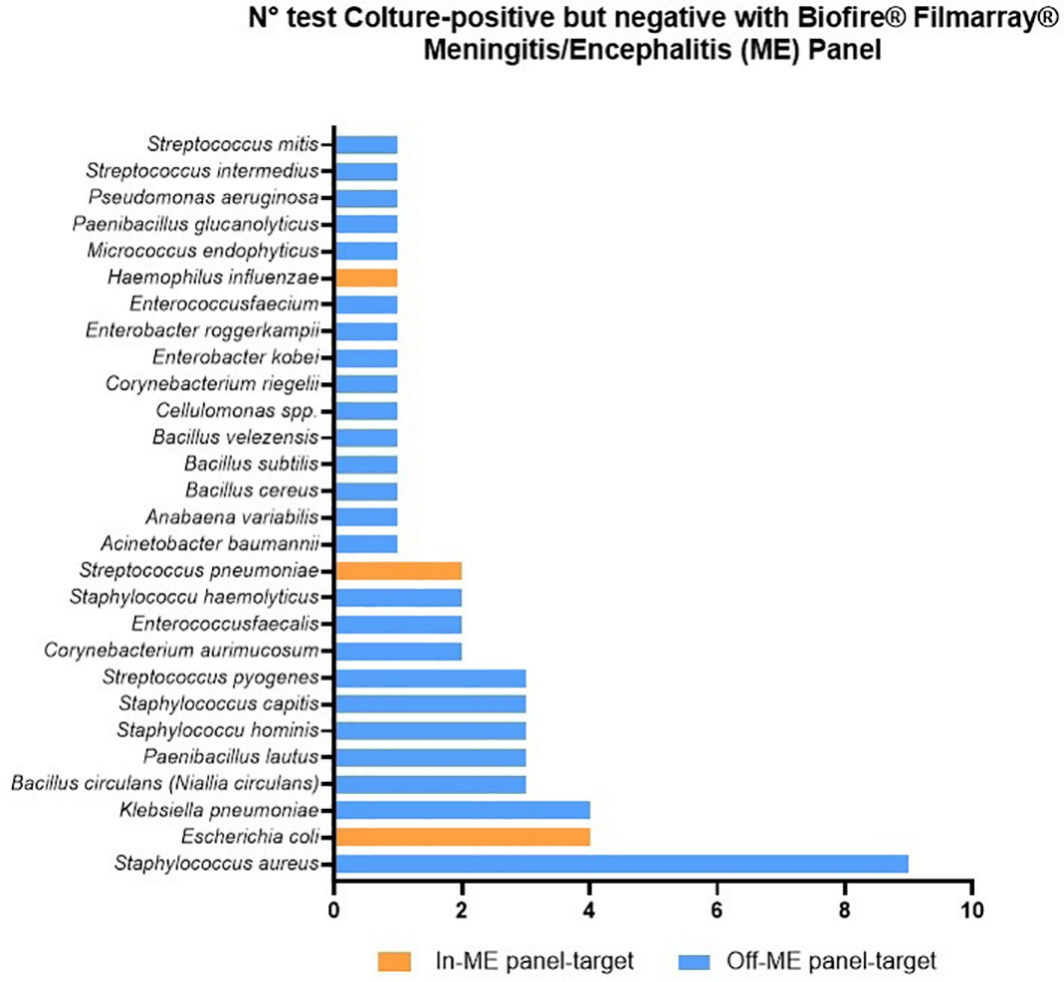
Distribution of culture−positive/BioFire^®^ FilmArray^®^ Meningitis/Encephalitis Panel−negative samples by pathogen.

The FA identified 183 viral infections, including Varicella zoster virus, Herpes simplex virus types 1 and 2, Human herpesvirus 6, Enterovirus, and Human Parechovirus. Among these viral episodes, only five samples yielded a positive bacterial culture. In all cases, the cultured organisms were bacteria not included in the FA panel, namely *Enterococcus faecalis*, *Staphylococcus aureus*, *Cellulosimicrobium* spp., *Micrococcus endophyticus*, and *Klebsiella pneumoniae*. When the analysis was restricted to bacterial targets only, the remaining 178 viral episodes were therefore classified as negative by both methods. Culture-positive isolates corresponding to FA off-panel bacterial pathogens (**Figure 1**), including *S. aureus*, *Enterococcus faecalis*, *Streptococcus pyogenes*, and *Cellulosimicrobium cellulans* (formerly *Cellulomonas cellulans*), were included from the core diagnostic accuracy and considered as double negative.

Conversely, culture−positive isolates corresponding to FA panel bacterial targets, namely *Escherichia coli K1* (n=4), *Haemophilus influenzae* (n=1), and *Streptococcus pneumoniae* (n=2), were retained in the concordance analysis and classified as bacterial false−negative FA results. After all these considerations, seven samples were identified as culture-positive and FA-negative (**Table 2**). Under this adjusted analytical framework, inter-method agreement increased substantially, with a Cohen’s κ coefficient of 0.736 (95% CI, 0.676–0.796). The FA demonstrated a sensitivity of 93.5% (95% CI, 87.0–97.3%) and a specificity of 97.6% (95% CI, 96.9–98.2%). The PPV was 62.5% (95% CI, 56.4–68.3%), while the NPV reached 99.7% (95% CI, 96.7–98.0%).

**Table 2:** Adjusted contingency table comparing BioFire^®^ FilmArray^®^ Meningitis/Encephalitis Panel (FA) and conventional culture results restricted to FA bacterial targets.

		Biofire^®^ Filmarray^®^ Meningitis/Encephalitis (ME) Panel		
		Positive	Negative	Total
Culture	Positive	100	7	107
	Negative	60	2419	2479
	Total	160	2426	2586

Values indicate the number of samples testing positive or negative by each method after exclusion of viral infections and bacterial pathogens not included in the FA panel.

## 4 Discussion

SPs have profoundly transformed the diagnostic approach to CNS infections by enabling rapid, standardized, and user-independent detection of predefined pathogens directly from CSF. Their implementation has improved early clinical decision-making, particularly in emergency and referral settings where timely results are essential to guide empiric antimicrobial therapy (Dien Bard and McElvania, 2020; Cassidy et al., 2021). This three-year real-world study confirms the utility of multiplex panels within an integrated workflow while also demonstrating that they remain insufficient, on their own, to deliver a complete and clinically robust etiological diagnosis. In line with these findings, recent real-world studies conducted in large referral cohorts have shown that syndromic meningitis/encephalitis panels, while highly specific, may still miss clinically relevant CNS infections, particularly when infections are caused by pathogens not included in the assay target list or are encountered in complex tertiary-care settings. Consistently, post-implementation evaluations have emphasized that the use of molecular panels as stand-alone tests may be insufficient, reinforcing the need for diagnostic stewardship approaches that formally integrate syndromic testing with conventional culture within structured diagnostic pathways (Erba et al., 2024; Sunnerhagen et al., 2024; Erinmez et al., 2026). This reinforces a diagnostic model in which molecular assays accelerate initial evaluation but do not replace the broader analytical spectrum provided by conventional microbiology.

A fundamental limitation of SP lies in their fixed and closed target architecture. Because they detect only organisms included in their predefined menu, clinically significant etiologies remain undetectable by design. In this study setting, conventional culture recovered several bacterial and fungal agents not represented in the panel, including *Staphylococcus aureus*, *Streptococcus pyogenes*, dimorphic fungi, and yeasts, all recognized causes of CNS infections, particularly in adults and immunocompromised patients (Ramanan et al., 2018; Kanjilal et al., 2019; Riddell and Wheat, 2019; van de Beek et al., 2021). This diagnostic gap becomes even more relevant in referral centers, where unusual, emerging or antimicrobial-pressure-modified infections occur with greater frequency. These observations underline the necessity of maintaining systematic culture in parallel with syndromic testing to ensure comprehensive pathogen recovery and to preserve phenotypic AST, which remains essential for targeted therapy and stewardship.

In addition to coverage limitations, multiple evaluations have documented variable and sometimes suboptimal sensitivity of multiplex molecular assays even for taxa included among their targets. Reduced sensitivity has been reported for *S. pneumoniae*, *H. influenzae*, *L. monocytogenes*, *N. meningitidis*, *S. agalactiae* and *E.coli* K1 (Ramanan et al., 2018; Tansarli and Chapin, 2020; López et al., 2024). These discrepancies are biologically plausible in scenarios of low bacterial burden, specimen heterogeneity or sampling after antimicrobial exposure, reinforcing the need for clinical-microbiological contextualization when interpreting negative panel results.

Conversely, positive molecular results with negative culture require careful interpretation. Numerous studies indicate that these discrepancies often represent true infections missed by culture, particularly in patients receiving prior antimicrobial therapy or with fastidious organisms (Dien Bard et al., 2016; Tansarli and Chapin, 2020; Vincent et al., 2020). The detection of residual antimicrobial activity in a consistent proportion of such cases in our cohort supports this interpretation and emphasizes that conventional culture, while essential, cannot always be considered an absolute reference standard. Accordingly, apparent molecular “false positives” should be adjudicated through an integrated framework that accounts for pre-analytical variables, antimicrobial exposure and pathogen characteristics, aligning with current diagnostic-stewardship recommendations.

Taken together, evidence from this study supports an integrated diagnostic strategy in which syndromic testing and conventional culture are systematically combined. SPs provide rapid, standardized and early actionable information, whereas culture remains indispensable for detecting off-panel organisms, resolving discordant results and performing phenotypic AST (Clinical breakpoint tables; Manual for the laboratory identification and antimicrobial susceptibility testing of bacterial pathogens of public health importance in the developing world; WHO). The identification of rare but clinically significant organisms such as *Cellulosimicrobium cellulans* further illustrates the added value of culture and reinforces its role in diagnostic completeness across multiple clinical scenarios (Ioannou et al., 2024; Narayan et al., 2024). From a system-level perspective, diagnostic algorithms, particularly in regional networks, should explicitly require parallel culture for all suspected CNS infections, ensuring that peripheral laboratories do not rely on panel-only pathways. Such an approach safeguards patient care and maintains the integrity of epidemiological and antimicrobial-resistance surveillance.

## 5 Study limitations

This study has several limitations that should be acknowledged. First, conventional culture was used as the primary reference standard for evaluating diagnostic performance, despite its well-recognized reduction in sensitivity in pre-treated patients and in infections characterized by low pathogen burden. Second, detailed clinical data (as well as cell count and gram stain information that were often performed at the peripheral hospital) were not systematically integrated into the evaluation of discordant panel and culture results, limiting the ability to definitively distinguish true infections from false-positive molecular detections or sample contamination. In addition, the lack of structured clinical data prevented age−stratified analyses, including separate evaluation of pediatric and adult populations, which is known to influence diagnostic performance in CNS infections. Third, this analysis represents a single−center real−world experience conducted in a tertiary referral setting, which may limit the generalizability of the findings to all clinical contexts. However, the institution’s role as a regional reference laboratory serving multiple peripheral hospitals enabled the inclusion of a diverse and heterogeneous sample. Together with the large sample size and the systematic use of parallel molecular and culture−based testing, this hub−and−spoke diagnostic model provides a robust analytical framework and supports the applicability of these findings to similar high−throughput diagnostic networks.

## 6 Conclusions

Although SPs have significantly improved the speed and standardization of CNS infection diagnostics, they cannot function as standalone diagnostic tools. Their optimal use requires systematic integration with conventional culture, which compensates for limitations in target coverage and analytical sensitivity while providing essential AST data. This integrated approach remains critical to ensure accurate, complete and clinically reliable diagnosis of CNS infections across diverse patient populations and healthcare settings. Future multiplex platforms should incorporate broader, adaptable and epidemiologically informed target architectures, while diagnostic pathways should continue to embed culture as an indispensable component of CNS infection workflows.

## Data Availability

The original contributions presented in the study are included in the article/supplementary material. Further inquiries can be directed to the corresponding authors.
